# Adaptation and Psychometric Evidence of the ARABIC Version of the Diabetes Self-Management Questionnaire (A-DSMQ)

**DOI:** 10.3390/healthcare10050951

**Published:** 2022-05-21

**Authors:** Nabil Kaddech, Noomen Guelmami, Tore Bonsaksen, Radhouene Doggui, Chiraz Beji, Jalila El Ati

**Affiliations:** 1 Nutritional Epidemiology and Surveillance Laboratory, National Institute of Nutrition and Food Technology, Tunis 1007, Tunisia; nabil10kaddech@yahoo.fr (N.K.); doggui.radhouene@gmail.com (R.D.); shiraz.beji@gmail.com (C.B.); jalila.elati@yahoo.fr (J.E.A.); 2 Department of Biology, University of Tunis El Manar, Tunis 1068, Tunisia; 3 Departement of Social Science, High Institute of Sport and Physical Education of Kef, Jendouba University, Kef 7100, Tunisia; 4 Department of Health Sciences (DISSAL), Postgraduate School of Public Health, University of Genoa, 16132 Genoa, Italy; 5 Department of Health and Nursing Science, Faculty of Social and Health Sciences, Inland Norway University of Applied Sciences, 2418 Elverum, Norway; tore.bonsaksen@inn.no; 6 Department of Health, Faculty of Health Studies, VID Specialized University, P.O. Box 184 Sandnes, Norway

**Keywords:** diabetes type 2, self-management, validity, reliability, physical activity

## Abstract

(1) Background: Diabetic patients must engage in self-care practices in order to maintain optimal glycemic control, hence reducing the likelihood of developing complications, and enhance the overall quality of their lives. The Diabetes Self-care Management Questionnaire (DSMQ) is a tool for assessing self-management habits that may be used to predict glycemic control in people with diabetes. However, no Arabic language version of the instrument has been found. Therefore, we adapted an Arabic language version of the instrument in Tunisia. The purpose of the current research aimed to assess the psychometric features of the Tunisian version of the DSMQ in patients with type 2 diabetes. (2) Method: Two samples including both genders, one exploratory (*n* = 208, mean age 53.2 ± 8.3) and one confirmatory (*n* = 441, mean age 53.4 ± 7.4), completed an adapted Arabic language version of the DSMQ, a sociodemographic questionnaire and information about their HbA1C levels. (3) Results: The exploratory factor analysis revealed that the 15 items of the A-DSMQ fit well with the data. Likewise, the alpha coefficients for the A-DSMQ factors were above 0.80: for “Glucose Management” (GM), “Dietary Control” (DC), “Physical Activity” (PA), and “Heath-Care Use” (HU). The fit indices for the CFA were good, and the four-factor solution was confirmed. The Average Variance Extracted values and Fornell–Larcker criterion established the convergent and discriminant validity, respectively. The concurrent validity of the tool was established through the statistically significant negative relationships between the A-DSMQ factors and HbA1C, in addition to its positive association with the practice of physical activity measured by the IPAQ. (4) Conclusions: Given the high EFA factor loadings, the CFA fit indices, the correlation matrix, the sensitivity analysis, the convergent validity, and the excellent internal consistency of the A-DSMQ, it can be concluded that the A-DSMQ is an effective psychometric tool for diabetes self-management in Tunisia.

## 1. Introduction

Diabetes is a chronic, complex metabolic health problem that can be considered one of the main causes of human mortality [1]. It generates serious consequences all over the world. It has a huge impact on the human physiology as well as cognitive, psychological and social functioning [2,3,4,5]. Diabetes has a vast societal and economic cost in terms of increased medical expenses, lost productivity and premature death, and intangible costs such as lower quality of life in many populations [6,7,8,9].

Globally, the prevalence of diabetes has reached 10.5% (536.6 million people), making it a worldwide public health concern [10]. Men and women have equal rates of diabetes, with those aged 75–79 years old having the highest rates. The age-adjusted diabetes prevalence in the world is 12.2 %, with the greatest incidence in Arab nations [11]. Type 2 diabetes (T2DM) is responsible for around 90 % of all diabetes cases in the world. More than 425 million people worldwide had diabetes in 2017, and according to current projections, this number is expected to rise to 629 million by 2045 [12]. Type 2 diabetes causes microvascular and macrovascular problems that give rise to significant psychological and physical discomfort in both patients and caregivers.

By preventing or delaying problems, early management of type 2 diabetes minimizes morbidity and mortality [13,14,15]. However, diabetes care is complicated and varied [16], since the condition is accompanied by a variety of complications and takes a considerable psychological toll on the person [17,18].

Effective diabetes care requires not just adequate euglycemic medication usage, but also patient education concerned with their medications, healthy dietary alternatives, exercise, and blood glucose self-monitoring [19,20,21].

Controlling glycated hemoglobin HbA1C helps regulate diabetes and decreases microvascular and macrovascular problems [22,23,24]. As it is the most significant long-term blood glucose control marker, the measurement and management of HbA1C is the gold standard of diabetes management [25]. Although many variables influence HbA1C, self-care behaviors such as blood sugar self-monitoring, a balanced diet, physical exercise, and a suitable medication regimen may help diabetes patients regulate their blood sugar levels [26]. Consequently, a standard questionnaire is required to assess self-care activities. A review of self-care questionnaires for diabetes patients revealed that most had unsatisfactory psychometric qualities and others examined just one component, such as medication adherence. Recent studies also found no consistent and significant relationships with HbA1c [27].

Numerous tools have been created to assess diabetic self-management behaviors. The Summary of Diabetes Self-Care Measures (SDSCA) is the most widely used and popular tool for assessing diabetes self-management practices [28,29]. On the other hand, the questionnaire was not created with glycated hemoglobin in mind, and a preliminary analysis indicated no significant connections between its scores and HbA1c [30].

The Diabetic Self-Management Questionnaire (DSMQ) developed by Schmitt et al. [30] is a new self-report tool for diabetes patients. The DSMQ has been shown to distinguish between patients with diabetes who are appropriately or inappropriately self-medicating, allowing for the early identification of patients at high risk for a bad diabetes prognosis [31]. The tool assesses four diabetic self-care domains: glucose regulation, food, exercise, and healthcare. The DSMQ is a simple [16-item] test that may be completed quickly. There was good psychometric fit when used in other language versions, such as German [30], Hungarian [32], Persian [28], Thai [33] and Urdu [34]. Additionally, a revised version has been validated in the Hungarian context [35]. Several investigations have also established links between diabetes self-management and HbA1C [36,37,38]. However, to our best knowledge, there is no appropriate Arabic version of the DSMQ to assess self-care in diabetic patients in Arabic countries.

Thus, this research attempted to adapt and psychometrically test the Arabic version of the DSMQ in a cohort of Tunisian T2DM patients.

## 2. Materials and Methods

### 2.1. Data Collection

A total of 800 subjects, who are regular patients at the National Institute of Nutrition and Food Technology in Tunisia, were invited to participate in this study. A total of 151 subjects who did not agree to participate in the study (*n* = 49) or who did not meet the inclusion criteria were excluded (*n* = 102). The final sample included 649 patients. Eligible participants had to be native Arabic speakers, over the age of 18, have had a T2DM diagnosis for at least two years, and be Tunisian. Participation in the study was not a requirement for drug treatment. Inability to complete the questionnaire was an exclusion criterion (for example, reading or comprehension problems). The recruited patients were randomly divided into exploratory (*n* = 208) and confirmatory samples (*n* = 441). Table 1 shows the socio-demographics of the samples. Participants gave their informed consent before the start of the survey.

### 2.2. Instruments

#### 2.2.1. Socio-Demographic Questionnaire and HbA1C

Participants provided data on age, gender, and socio-economic level (the minimum wage, between two and three times the minimum wage, and more than three times the minimum wage). Academic level was coded as graduate and undergraduate, and the Other Disease was coded binary (Yes or No). Patients’ HbA1c (glycated hemoglobin) was quantified by electrophoresis on a Capillarys 2 Flex Piercing apparatus at the laboratory of the National Institute of Nutrition and Food Technology in Tunisia as the average glycaemia of the last three months and presented as the percentage of hemoglobin that has bound sugar in the blood.

#### 2.2.2. Arabic Version of the Diabetes Self-Management Questionnaire (A-DSMQ)

To assess diabetes self-management, we used an Arabic adapted version of the Diabetes Self-Management Questionnaire (DSMQ). The original self-report questionnaire consisted of four subscales evaluated with 15 items, which were “Glucose Management” (GM), “Dietary Control” (DC), “Physical Activity” (PA), and “Heath-Care Use” (HU), as well as one additional item 16, which must be included in the “Sum Scale” (SS), that served as an overall assessment of self-care [28]. The initial scale exhibited good reliability (‘Sum Scale’ α coefficient = 0.84) and validity (CFI = 0.95 and RMSEA = 0.053) [39].

#### 2.2.3. International Physical Activity Questionnaire (IPAQ) 

The Physical activity level was quantified using the IPAQ’s approved Arabic short version [40]. The IPAQ short form utilized in this investigation has seven items that assess time spent walking, engaging in vigorous- and moderate-intensity physical activities, and engaging in sedentary activity over the preceding seven days. IPAQ defines moderate physical activity as any activity that results in a modest rise in respiratory rate, heart rate, and perspiration for a minimum of ten minutes. This equates to 3–6 metabolic equivalents (MET) based on the physical activity compendium. Vigorous physical activities are those that result in a sharp rise in respiratory rate, heart rate, and perspiration for at least ten minutes. The metabolic equivalent is greater than six MET [40].

### 2.3. Procedure

Two committees (each with two translators and bilingual clinicians) performed forward and backward translations of the A-DSMQ.

Forward translation: Two independent English–Arabic bilingual translators (bilingual clinician and licensed translator) translated the original English scales into two Arabic drafts. The first consensus method involved an independent multilingual person comparing both Arabic drafts to each other as well as the original English version. This comparison sought to identify and resolve any uncertainties and differences in the meanings of words or sentences. Any non-agreed items, phrases, or words during this comparison were discussed and revised by both translators, the first author, and the independent bilingual person.

Back translation: The Arabic version was back translated to English by two English–Arabic bilingual independent translators (a bilingual clinician and a licensed translator) who were unaware of the forward translation technique and the original English versions of the scales.

Finally, a small group of both men and women (*n* = 30) were requested to complete the Arabic version of the scale as a pre-test.

Ethical Approval: Institutional review board approval for the cohort study was obtained from both the Tunisian National Institute of Nutrition (Tunis, Tunisia) and the University of Jendouba Research Ethics Committee.

### 2.4. Statistical Analysis

The analyses were performed using SPSS 26.0.00 (IBM Inc., Chicago, IL, USA) and Lavaan package in RStudio free Software (Boston, USA). For age, education, and reported income, descriptive statistics, (means with standard deviations and frequency distributions) were produced.

Skewness and kurtosis tests were used to determine data normality during the exploratory phase, whereas multivariate normality was assessed during the confirmatory phase. Skewness values greater than 7 or kurtosis values greater than 3 were considered non-Gaussian and associated with low psychometric sensitivity. Additionally, during the confirmatory phase, the Mardia coefficient of multivariate normality was determined.

The factor structure was originally validated using principal component analysis (PCA) and varimax rotation with Kaiser Normalization. More precisely, varimax rotation was chosen above other types of rotation because, unlike the others, it permits the reduction in component complexity while increasing the variation in factor loadings [41].

The probable number of factors was determined by computing the number of factors with eigenvalues greater than one [41,42] and visually evaluating the Cattell’s scree-plot. After examining the factor loadings, entries with unacceptable loadings (that is, values < 0.3) were removed. Additionally, elements were excluded and suppressed if their factor loading contradicted a reasonable theoretical explanation.

Following that, confirmatory factor analysis (CFA) was performed. Numerous scholars have suggested and recommended calculating and reporting a variety of fit indices, including the following: (i) discrepancy indices (including chi-squared and Steiger–Lind’s root-mean-square error of approximation (RMSEA), and (ii) tests comparing the target model to the null model, such as the Bentler–normed Bonett’s fit index (NFI), which is calculated by dividing the chi-squared value by the degrees of freedom (df) value. According to Schumacker and Lomax [43], RMSEA values greater than 0.10 indicate unsuitable models [44], while TLI and CFI should be greater than 0.95 [45]. Finally, the GFI and AGFI cut-off and threshold values should be greater than 0.90 [45].

Convergent validity was determined using the average variance extracted (AVE) and the Fornell–Larcker criterion, respectively [46]. AVE levels more than 0.7 are considered highly good, while values less than 0.5 are deemed acceptable [47]. When the variance shared by two distinct latent variables is smaller than the variance shared by the latent variable and its indicators, discriminant validity is assured (i.e., items). This indicates that the square root of the AVE must be bigger than the sum of all latent variable correlations [48].

## 3. Results

### 3.1. Descriptive Statistics and PCA

The descriptive statistics in Table 2 include the means and standard deviations; the normality skewness and kurtosis coefficients; and the lambda factor loadings. The normality coefficients substantiate the normality of the distributions.

**Figure 1:**
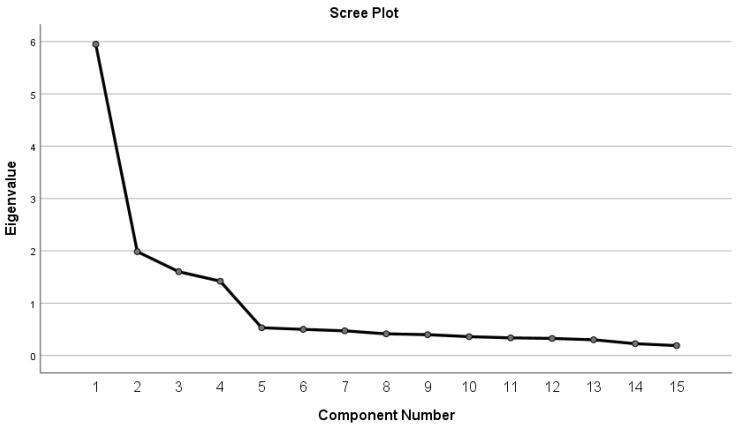
Scree plot of the Arabic version of Diabetes Self-Management Questionnaire (A-DSMQ).

The results show that the A-DSMQ is suitable for factor analysis (KMO = 0.87; Bartlett sphericity test = 1649.07; df = 105; *p* < 0.001). PCA revealed a four-factor solution (eigenvalues of 5.95, 1.99, 1.60 and 1.42 for the first, second, third and fourth factors, respectively), accounting for up to 73.03% of total variance and with lambda factor loadings ranging from 0.72 to 0.87 for the items. The first component accounted for 39.70% of total variance, the second factor accounted for 13.23% of the variance, the third factor accounted for 10.98% of the total variance and the fourth factor 9.46% of the total variance. Additionally, the scree plot validates the four-factor approach; a clear shift in the slope is visible in Figure 1.

### 3.2. Reliability

The internal consistency and reliability of the four scale factors were examined by the Cronbach’s α, coefficient (Table 3). Examination of the reliability indices for the four components of the scale yielded values greater than or equal to 0.80. This provides evidence for the internal consistency of the scale. In addition, the corrected item-total correlation was calculated for each latent variable. The results show that the values were adequate, since they were located between 0.738 and 0.823 for the Glucose Management (GM), between 0.710 and 0.729 for the Dietary Control (DC), between 0.613 and 0.736 for the Heath-Care Use (HU), and between 0.650 and 0.743 for the Physical Activity (PA). The internal consistency of the component is considered good if the value is equal to or greater than 0.70. The 15 items of A-DSMQ total score exhibited a 0.89 Cronbach’s Alpha value. These results confirm that the instrument has good reliability.

### 3.3. Confirmatory Factor Analysis

**Figure 2:**
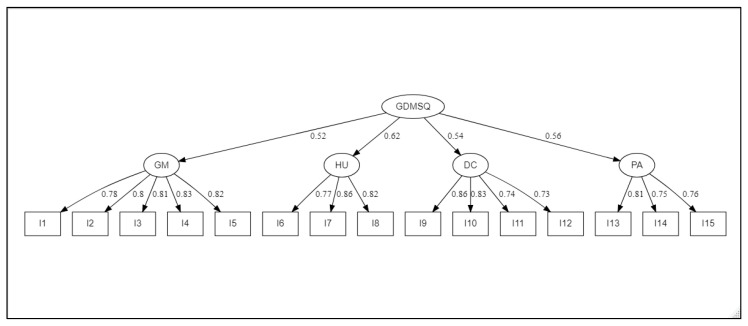
The final confirmatory factor analysis [CFA] of A-DSMQ. Footnotes: CFA statistics: χ^2^_(89)_ = 99.89, *p* = *0*.20; χ^2^/df = 1.47; GFI = 0.998; AGFI = 0.991; TLI = 0.998; CFI = 0.998; RMSEA = 0.017 [90% CI 0–0.032]; SRMR = 0.043. GM = Glucose Management, DC = Dietary Control, PA = Physical Activity and HU = Heath-Care Use.

We used the robust Maximum Likelihood estimation approach to perform confirmatory factorial analysis (CFA) of the first and second order. Psychometric data are always non-normal in their multivariate distribution. Thus, we used the Mardia coefficient to determine the multivariate normality of the data (Table 4). The findings indicated that the distribution was not normal (Multivariate Kurtosis = 4.31; Kurtosis Critical values C.R_k_ = 2.01), while the indices generated by the CFA indicated a conformance factorial structure that is compatible with the initial model. A product’s resilience increases as its factor loadings increase. The current research found that the second-order confirmatory factorial analysis of the 15 items had good factorial loadings ranging from 0.73 to 0.86 (see Figure 2).

The model’s fit indices values were suitable: the chi-square/degree of freedom (χ^2^ /df) = 1.47, the goodness of fit index (GFI = 0.998), the goodness of fit index (AGFI = 0.991), the comparative fit index (CFI = 0.998) and The Tucker–Lewis index (TLI = 0.998). Likewise, the errors fit index was adequate: the root mean square error of approximation (RMSEA = 0.017), and the standardized root mean square residual were investigated (SRMR = 0.043). Typically, values of RMSEA less than 0.08, and SRMR less than 0.08 are advised to demonstrate a reasonable fit to the data.

### 3.4. Concurrent Validity of the A-DSMQ

Statistically significant negative relationships were found between the A-DSMQ factors and HbA1C, as revealed by the correlation matrix. Table 5 shows the correlations between the variables. Furthermore, with regard to the Pearson correlation table, the correlation between GM and HbA1C was the highest value (r = −0.41). While the HU was highly correlated with the HbA1C (r = −0.23), a high correlation that was significant at the 0.001 level); the DC factor of the A-DSMQ was associated with HbA1C (r = −0.28). Finally, the Pearson correlation coefficient between PA and HbA1C was −0.36 and indicated a moderate correlation, while the total score exhibited the highest correlation level (r = −0.48) and indicated a high correlation with the HbA1C.

### 3.5. Convergent and Discriminant Validity

The average variance extracted AVE was 0.65, 0.67, 0.63 and 0.60 for Glucose Management (GM), Dietary Control (DC), Heath-Care Use (HU) and Physical Activity (PA), respectively. These values establish the convergent validity of the scale. The square root of the AVE values: 0.81, 0.82, 0.79 and 0.77 for GM, HU DC and PA, respectively, were greater than all correlations between the factors of the A-DSMQ. This result confirms the discriminant validity of the scale.

## 4. Discussion

This research aimed to investigate the psychometric features of an Arabic language adaptation of the Diabetes Self-Management Questionnaire [DSMQ]. The Arabic version of the DSMQ, abbreviated A-DSMQ, strengthened the presence of four components, as indicated by 15 questions. In terms of the reliability of the adapted tool, the Cronbach’s alpha coefficient demonstrates that the internal consistency of the four resulting scales is satisfactory. Moreover, confirmatory factor analysis suggested that the structure of the scales fit well the data and confirmed the DSMQ initial structure. In a related study Schmitt et al. [39] showed that the self-management and glycemic control structural equation models fit the data extremely well. Self-management, as measured by the DSMQ, was associated with HbA1c of –0.53 for type 1 and –0.46 for type 2 diabetes, respectively, accounting for 21% and 28% of variation in glycemic control, respectively. Similar to our study, Mirzaei et al. [28], through EFA with Varimax rotation, extracted four components that accounted for 67.36% of the total variance of the Persian version of the DSMQ scale. Moreover, the CFA, which demonstrated a reasonable fit for the four-factor structure retrieved from the EFA [CFI: 0.98, TLI: 0.97, RMSEA: 0.042], are as follows: Internal consistency was high: Cronbach’s alpha coefficient for the whole scale was 0.84; for the subscales, it ranged from 0.75 to 0.91 [28]. The convergent validity analysis revealed substantial negative correlations between DSMQ subscales and HbA1c.

Equivalent results were reported on the reliability of the Urdu version [34]. All, DSMQ scales and the total score had a high degree of internal consistency. For consideration of factor structure, after modeling all significant correlations between the items’ error terms consecutively, an adequate fit to the data was obtained for the single factor model (TLI = 0.98, CFI = 0.99, and RMSEA = 0.05). In addition, the first order four factor model has significantly worse fit indices to the data, whereas a higher order factorial structure has not been examined. Similarly, concurrent validity showed significant strong negative associations with HbA1c. In contrast, except for the physical activity subscale, the Hungarian version of DSMQ [32] had minimal to fair association with HbA1c. A negative relationship (r = 0.25) was discovered between the DSMQ sum scale and HbA1c values. There was a significant slight correlation between HbA1c and subscales of glucose management (r = 0.16) and nutritional control (r = 0.23). As for our study, research has shown associations between HbA1c and the practice of physical activity [49,50].

### Limitation of the Study

One of the study’s drawbacks is its cross-sectional design and the limited representativeness of the sample size at the national level, since the majority of the participants lived in Tunisia’s metropolitan areas. In addition, another limitation of this study is the respondents’ unknown literacy rate.

## 5. Conclusions

This study demonstrates that the A-DSMQ has reliability and validity in Tunisia patients with type 2 diabetes.

While further research is needed to assess the self-management of type 2 diabetes and its social determinants in a larger and more diverse Tunisian population, the validated A-DSMQ have proven to be reliable and valid for use in the clinical setting in Tunisia. Further research is required in the rest of the Arab world to determine whether the tool, if adapted to each country’s health system, can be used to measure the management of type 2 diabetes at the local level.

## Figures and Tables

**Table 1:** Demographics of samples.

Variables		Exploratory (*n* = 208)		Confirmatory (*n* = 441)	
		*n*	%	*n*	%
Gender	Female	121	58.17%	169	38.32%
	Male	87	41.83%	272	61.68%
Socio economic level	Low	67	32.21%	117	26.53%
	Medium	80	38.46%	220	49.89%
	High	61	29.33%	104	23.58%
Academic level	Graduate	87	41.83%	224	50.79%
	Undergraduate	121	58.17%	217	49.21%
Other disease	Yes	31	14.90%	52	11.79%
	No	177	85.10%	389	88.21%
Age	Mean ± Std. Deviation	53.2 ± 8.2		53.4 ± 7.4	
HbA1c value (%)	Mean ± Std. Deviation	8.2 ± 1.3		8.6 ± 1.7	

**Table 2:** Descriptive statistics of the exploratory sample (*n* = 208).

	Mean	Std. Deviation	Skewness	Kurtosis	Lambda
I1	1.70	1.03	−0.33	−1.02	0.86
I2	1.76	0.98	−0.31	−0.90	0.81
I3	1.70	1.00	−0.33	−0.93	0.80
I4	1.83	0.85	−0.20	−0.70	0.82
I5	1.78	0.82	−0.27	−0.42	0.80
I6	1.91	0.82	−0.20	−0.74	0.78
I7	1.74	0.90	−0.29	−0.66	0.86
I8	1.70	0.97	−0.22	−0.94	0.87
I9	1.79	0.76	−0.43	0.06	0.83
I10	1.78	0.80	−0.59	0.10	0.79
I11	1.80	0.80	−0.25	−0.38	0.72
I12	1.77	0.82	−0.52	−0.05	0.79
I13	1.77	0.85	−0.24	−0.57	0.77
I14	1.70	0.88	−0.35	−0.52	0.84
I15	1.77	0.90	−0.34	−0.61	0.85

**Table 3:** Internal consistency of the Arabic version of Diabetes Self-Management Questionnaire (A-DSMQ).

Factors	Items	Scale Mean if Item Deleted	Corrected Item-Total Correlation	Cronbach’s Alpha if Item Deleted	Cronbach’s Alpha
**GM**	I1	7.08	0.823	0.869	0.904
	I2	7.01	0.749	0.885	
	I3	7.07	0.769	0.881	
	I4	6.94	0.739	0.888	
	I5	6.99	0.738	0.889	
**DC**	I6	3.43	0.710	0.807	0.851
	I7	3.61	0.729	0.785	
	I8	3.64	0.736	0.783	
**HU**	I9	5.36	0.692	0.768	0.828
	I10	5.37	0.637	0.791	
	I11	5.35	0.613	0.802	
	I12	5.38	0.680	0.772	
**PA**	I13	3.47	0.650	0.822	0.838
	I14	3.54	0.743	0.733	
	I15	3.47	0.711	0.765	

**Table 4:** Descriptive statistics, univariate and multivariate normality of items at confirmatory phase.

Variable	Mean	Std. Deviation	Skew	C.R_S_	Kurtosis	C.R_k_
I1	1.82	1.04	−0.36	−3.12	−1.08	−4.63
I2	1.83	0.99	−0.30	−2.58	−1.02	−4.36
I3	1.79	0.99	−0.35	−2.97	−0.92	−3.92
I4	1.74	1.04	−0.35	−2.96	−1.04	−4.45
I5	1.80	0.97	−0.39	−3.35	−0.82	−3.52
I6	1.91	0.89	−0.53	−4.52	−0.41	−1.76
I7	1.88	0.88	−0.31	−2.67	−0.72	−3.08
I8	1.85	0.97	−0.28	−2.43	−1.01	−4.31
I9	1.88	0.90	−0.36	−3.06	−0.74	−3.15
I10	1.89	0.94	−0.32	−2.75	−0.94	−4.04
I11	1.79	0.97	−0.39	−3.38	−0.82	−3.52
I12	1.79	0.96	−0.37	−3.19	−0.81	−3.45
I13	1.90	0.93	−0.61	−5.22	−0.41	−1.77
I14	2.00	0.93	−0.52	−4.43	−0.73	−3.11
I15	1.88	0.98	−0.39	−3.30	−0.95	−4.06

Footnote: C.R_S_ = Skew Critical values; C.R_k_; C.R_k_ = Kurtosis Critical values.

**Table 5:** Correlation matrix between A-DSMQ factors, total score and HbA1C.

	GM	HU	DC	PA	A-DSMQ	HbA1C
HU	0.32 **					
DC	0.25 **	0.23 **				
PA	0.26 **	0.22 **	0.31 **			
SS	0.76 **	0.59 **	0.66 **	0.60 **		
HbA1C	−0.41 **	−0.23 **	−0.28 **	−0.36 **	−0.49 **	
IPAQ	0.23 **	0.17 **	0.17 **	0.47 **	0.38 **	−0.27 **

** Correlation is significant at the 0.01 level.

## Data Availability

The datasets used and/or analyzed during the current study are available from the corresponding author on reasonable request.

## References

[1] Takashi Y., Kawanami D. (2022). The Role of Bone-Derived Hormones in Glucose Metabolism, Diabetic Kidney Disease and Cardiovascular Disorders. Int. J. Mol. Sci..

[2] O’Toole S.M., Walker R.J., Garacci E., Dawson A.Z., Campbell J.A., Egede L.E. (2022). Explanatory role of sociodemographic, clinical, behavioral, and social factors on cognitive decline in older adults with diabetes. BMC Geriatr..

[3] Niroomand M., Babaniamansour S., Aliniagerdroudbari E., Golshaian A., Meibodi A.M., Absalan A. (2021). Distress and depression among patients with diabetes mellitus: Prevalence and associated factors: A cross-sectional study. J. Diabetes Metab. Disord..

[4] Saad A.M., Younes Z., Abuali A., Farooqi M.H., Hassoun A.A. (2021). Diabetes distress and depression among patients with type 2 diabetes: A cross-sectional study. J. Diabetes Endocr. Pract..

[5] Twiddy H., Frank B., Alam U. (2021). A consideration of the psychological aspects to managing patients with painful diabetic neuropathy: An insight into pain management services at a tertiary centre in the UK. Diabetes Ther..

[6] Ding, C.; Bao, Y.; Bai, B.; Liu, X.; Shi, B.; Tian, L. An update on the economic burden of type 2 diabetes mellitus in China. *Expert Rev. Pharm. Outcomes Res.* 2022, *accepted*.

[7] Ebrahimipour H., Keyvanlo Z., Heidarian Miri H., Yousefi M., Ariafar M., Rezazadeh A., Pourahmadi E. (2021). Productivity Loss of Diabetes in Iran (South Khorasan Province). J. Res. Health.

[8] Ganasegeran K., Hor C.P., Jamil M.F.A., Loh H.C., Noor J.M., Hamid N.A., Suppiah P.D., Abdul Manaf R.D., Hock Ch’ng A.S., Looi I. (2020). A systematic review of the economic burden of type 2 diabetes in Malaysia. Int. J. Environ. Res. Public Health.

[9] American Diabetes Association (2018). Economic costs of diabetes in the US in 2017. Diabetes Care.

[10] Sun H., Saeedi P., Karuranga S., Pinkepank M., Ogurtsova K., Duncan B.B., Stein C., Basit A., Chan J.C.N., Mbanya J.C. (2022). IDF Diabetes Atlas: Global, Regional and Country-Level Diabetes Prevalence Estimates for 2021 and Projections for 2045. Diabetes Res. Clin. Pract..

[11] El-Kebbi I.M., Bidikian N.H., Hneiny L., Nasrallah M.P. (2021). Epidemiology of type 2 diabetes in the Middle East and North Africa: Challenges and call for action. World J. Diabetes.

[12] Forouhi N.G., Wareham N.J. (2019). Epidemiology of diabetes. Medicine.

[13] Khor S.M., Choi J., Won P., Ko S.H. (2022). Challenges and Strategies in Developing an Enzymatic Wearable Sweat Glucose Biosensor as a Practical Point-Of-Care Monitoring Tool for Type II Diabetes. Nanomaterials.

[14] Mallik R., Chowdhury T.A. (2022). Pharmacotherapy to delay the progression of diabetic kidney disease in people with type 2 diabetes: Past, present and future. Ther. Adv. Endocrinol. Metab..

[15] Gong Q., Zhang P., Wang J., Ma J., An Y., Chen Y., Zhang B., Feng X., Li H., Chen X. (2019). Morbidity and mortality after lifestyle intervention for people with impaired glucose tolerance: 30-year results of the Da Qing Diabetes Prevention Outcome Study. Lancet Diabetes Endocrinol..

[16] Mechanick J.I., Adams S., Davidson J.A., Fergus I.V., Galindo R.J., McKinney K.H., Petak S.M., Sadhu A.R., Samson S.L., Vedanthan R. (2019). Transcultural diabetes care in the United States—A position statement by the American Association of Clinical Endocrinologists. Endocr. Pract..

[17] Wylie T.A.F., Shah C., Connor R., Farmer A.J., Ismail K., Millar B., Morris A., Reynolds R.M., Robertson E., Swindell R. (2019). Transforming mental well-being for people with diabetes: Research recommendations from Diabetes UK’s 2019 Diabetes and Mental Well-Being Workshop. Diabet. Med..

[18] Kalra S., Jena B.N., Yeravdekar R. (2018). Emotional and psychological needs of people with diabetes. Indian J. Endocrinol. Metab..

[19] Mathews E., Sathish T., Joseph A., Kodapally B., Thulaseedharan J.V., Narayan K.V., Oldenburg B., Thankappan K.R. (2022). Effectiveness and implementation of a lifestyle modification intervention for women with isolated impaired fasting glucose: Study protocol for a hybrid type 2 study in Kerala, India. Wellcome Open Res..

[20] Lind N., Hansen D.L., Rasmussen S.S., Nørgaard K. (2021). Real-time continuous glucose monitoring versus self-monitoring of blood glucose in adults with insulin-treated type 2 diabetes: A protocol for a randomised controlled single-centre trial. BMJ Open.

[21] Weinstock R.S., Aleppo G., Bailey T.S., Bergenstal R.M., Fisher W.A., Greenwood D.A., Young L.A. (2021). The role of blood glucose monitoring in diabetes management. PMC.

[22] Fan E.Y., Crawford A.S., Nguyen T., Judelson D., Learned A., Chan J., Schanzer A., Simons J.P., Jones D.W. Hemoglobin A1C Monitoring Practices Prior to Lower Extremity Bypass in Patients with Diabetes Vary Broadly and Do Not Predict Outcomes. J. Vasc. Surg..

[23] Shah N.A., Levy C.J. (2021). Emerging technologies for the management of type 2 diabetes mellitus. J. Diabetes.

[24] Martinez M., Santamarina J., Pavesi A., Musso C., Umpierrez G.E. (2021). Glycemic variability and cardiovascular disease in patients with type 2 diabetes. BMJ Open Diabetes Res. Care.

[25] Gomez-Peralta F., Choudhary P., Cosson E., Irace C., Rami-Merhar B., Seibold A. (2022). Understanding the clinical implications of differences between GMI and HbA1c. Diabetes Obes. Metab..

[26] Akalin S., Berntorp K., Ceriello A., Das A.K., Kilpatrick E.S., Koblik T., Munichoodappa C.S., Pan C.Y., Rosenthall W., Shestakova M. (2009). Intensive glucose therapy and clinical implications of recent data: A consensus statement from the Global Task Force on Glycaemic Control. Int. J. Clin. Pract..

[27] Lu Y., Xu J., Zhao W., Han H.-R. (2016). Measuring self-care in persons with type 2 diabetes: A systematic review. Eval. Health Prof..

[28] Mirzaei H., Siavash M., Shahnazi H., Abasi M.H., Eslami A.A. (2022). Assessment of the psychometric properties of the Persian version of the diabetes self-management questionnaire (DSMQ) in patients with type 2 diabetes. J. Diabetes Metab. Disord..

[29] Kong S.-Y., Cho M.-K. (2021). Validity and Reliability of the Korean Version of the Self-Care of Diabetes Inventory (SCODI-K). Int. J. Environ. Res. Public Health.

[30] Schmitt A., Gahr A., Hermanns N., Kulzer B., Huber J., Haak T. (2013). The Diabetes Self-Management Questionnaire (DSMQ): Development and evaluation of an instrument to assess diabetes self-care activities associated with glycaemic control. Health Qual. Life Outcomes.

[31] Schmitt A., Hermanns N., Kulzer B., Reimer A., Schall S., Haak T. (2014). The Diabetes Self-Management Questionnaire (DSMQ) can detect inadequate self-care behaviour and help identify patients at risk of a negative diabetes prognosis. Diabetologia.

[32] Vincze A., Losonczi A., Stauder A. (2020). The validity of the diabetes self-management questionnaire (DSMQ) in Hungarian patients with type 2 diabetes. Health Qual. Life Outcomes.

[33] Thojampa S., Mawn B. (2017). Psychometric evaluation of the Thai translation of the Diabetes Self-management Questionnaire in type 2 diabetes. Int. J. Nurs. Sci..

[34] Bukhsh A., Lee S.W.H., Pusparajah P., Schmitt A., Khan T.M. (2017). Psychometric properties of the diabetes self-management questionnaire (DSMQ) in Urdu. Health Qual. Life Outcomes.

[35] Márkus B., Hargittay C., Iller B., Rinfel J., Bencsik P., Oláh I., Kalabay L., Vörös K. (2022). Validation of the revised Diabetes Self-Management Questionnaire (DSMQ-R) in the primary care setting. BMC Prim. Care.

[36] Bekele B.B., Negash S., Bogale B., Tesfaye M., Getachew D., Weldekidan F., Balcha B. (2021). Effect of diabetes self-management education (DSME) on glycated hemoglobin (HbA1c) level among patients with T2DM: Systematic review and meta-analysis of randomized controlled trials. Diabetes Metab. Syndr. Clin. Res. Rev..

[37] Perlman J.E., Gooley T.A., McNulty B., Meyers J., Hirsch I.B. (2021). HbA1c and glucose management indicator discordance: A real-world analysis. Diabetes Technol. Ther..

[38] Beran M., Muzambi R., Geraets A., Albertorio-Diaz J.R., Adriaanse M.C., Iversen M.M., Kokoszka A., Nefs G., Nouwen A., Pouwer F. (2022). The bidirectional longitudinal association between depressive symptoms and HbA_1c_: A systematic review and meta-analysis. Diabet. Med..

[39] Schmitt A., Reimer A., Hermanns N., Huber J., Ehrmann D., Schall S., Kulzer B. (2016). Assessing diabetes self-management with the diabetes self-management questionnaire (DSMQ) can help analyse behavioural problems related to reduced glycaemic control. PLoS ONE.

[40] Al-Hazzaa H.M. (2007). Health-enhancing physical activity among Saudi adults using the International Physical Activity Questionnaire (IPAQ). Public Health Nutr..

[41] Tabachnick B.G., Fidell L.S., Ullman J.B. (2007). Using Multivariate Statistics.

[42] Fidell A. (2009). Discovering Statistics with SPSS.

[43] Schumacker R.E., Lomax R.G. (2004). A Beginner’s Guide to Structural Equation Modeling.

[44] Steiger J.H. (2000). Point estimation, hypothesis testing, and interval estimation using the RMSEA: Some comments and a reply to Hayduk and Glaser. Struct. Equ. Modeling.

[45] Hu L., Bentler P.M. (1999). Cutoff criteria for fit indexes in covariance structure analysis: Conventional criteria versus new alternatives. Struct. Equ. Modeling Multidiscip. J..

[46] Cheung G.W., Wang C. (2017). Current approaches for assessing convergent and discriminant validity with SEM: Issues and solutions. Academy of Management Proceedings.

[47] Aimran A.N., Ahmad S., Afthanorhan A., Awang Z. (2017). The assessment of the performance of covariance-based structural equation modeling and partial least square path modeling. AIP Conf. Proc..

[48] Ab Hamid M.R., Sami W., Sidek M.M. (2017). Discriminant validity assessment: Use of Fornell & Larcker criterion versus HTMT criterion. J. Phys. Conf. Ser..

[49] Yun I., Joo H.J., Park Y.S., Park E.-C. (2022). Association between Physical Exercise and Glycated Hemoglobin Levels in Korean Patients Diagnosed with Diabetes. Int. J. Environ. Res. Public Health.

[50] Yanai H., Adachi H., Masui Y., Katsuyama H., Kawaguchi A., Hakoshima M., Waragai Y., Harigae T., Hamasaki H., Sako A. (2018). Exercise therapy for patients with type 2 diabetes: A narrative review. J. Clin. Med. Res..

